# Biopesticides as a promising alternative to synthetic pesticides: A case for microbial pesticides, phytopesticides, and nanobiopesticides

**DOI:** 10.3389/fmicb.2023.1040901

**Published:** 2023-02-16

**Authors:** Modupe S. Ayilara, Bartholomew S. Adeleke, Saheed A. Akinola, Chris A. Fayose, Uswat T. Adeyemi, Lanre A. Gbadegesin, Richard K. Omole, Remilekun M. Johnson, Qudus O. Uthman, Olubukola O. Babalola

**Affiliations:** ^1^Food Security and Safety Focus Area, Faculty of Natural and Agricultural Sciences, North-West University, Mmabatho, South Africa; ^2^Department of Biological Sciences, Kings University, Ode-Omu, Nigeria; ^3^Department of Biological Sciences, Microbiology Unit, School of Science, Olusegun Agagu University of Science and Technology, Okitipupa, Nigeria; ^4^Department of Microbiology and Parasitology, School of Medicine and Pharmacy, College of Medicine and Health Sciences, University of Rwanda, Butare, Rwanda; ^5^Department of Agricultural Technology, Ekiti State Polytechnic, Isan-Ekiti, Nigeria; ^6^Department of Agricultural Economics and Farm Management, Faculty of Agriculture, University of Ilorin, Ilorin, Nigeria; ^7^Institute of Mountain Hazards and Environment, University of Chinese Academy of Sciences, Chengdu, China; ^8^Department of Microbiology, Obafemi Awolowo University, Ile-Ife, Nigeria; ^9^Microbiology Unit, Department of Applied Sciences, Osun State College of Technology, Esa-Oke, Nigeria; ^10^Soil, Water and Ecosystem Sciences, University of Florida, Gainesville, FL, United States

**Keywords:** nanoparticles, biopesticides, synthetic pesticides, soil health, pesticides

## Abstract

Over the years, synthetic pesticides like herbicides, algicides, miticides, bactericides, fumigants, termiticides, repellents, insecticides, molluscicides, nematicides, and pheromones have been used to improve crop yield. When pesticides are used, the over-application and excess discharge into water bodies during rainfall often lead to death of fish and other aquatic life. Even when the fishes still live, their consumption by humans may lead to the biomagnification of chemicals in the body system and can cause deadly diseases, such as cancer, kidney diseases, diabetes, liver dysfunction, eczema, neurological destruction, cardiovascular diseases, and so on. Equally, synthetic pesticides harm the soil texture, soil microbes, animals, and plants. The dangers associated with the use of synthetic pesticides have necessitated the need for alternative use of organic pesticides (biopesticides), which are cheaper, environment friendly, and sustainable. Biopesticides can be sourced from microbes (e.g., metabolites), plants (e.g., from their exudates, essential oil, and extracts from bark, root, and leaves), and nanoparticles of biological origin (e.g., silver and gold nanoparticles). Unlike synthetic pesticides, microbial pesticides are specific in action, can be easily sourced without the need for expensive chemicals, and are environmentally sustainable without residual effects. Phytopesticides have myriad of phytochemical compounds that make them exhibit various mechanisms of action, likewise, they are not associated with the release of greenhouse gases and are of lesser risks to human health compared to the available synthetic pesticides. Nanobiopesticides have higher pesticidal activity, targeted or controlled release with top-notch biocompatibility and biodegradability. In this review, we examined the different types of pesticides, the merits, and demerits of synthetic pesticides and biopesticides, but more importantly, we x-rayed appropriate and sustainable approaches to improve the acceptability and commercial usage of microbial pesticides, phytopesticides, and nanobiopesticides for plant nutrition, crop protection/yield, animal/human health promotion, and their possible incorporation into the integrated pest management system.

## 1. Introduction

From antiquity, the use of synthetic (chemical) pesticides to control crop pests for improved crop production is known (Anani et al., 2020). Synthetic pesticides are made from chemicals and carriers, such as polymers (Rakhimol et al., 2020), which are specific for different pests. They range from those employed in the control of weeds (herbicides), algae (algicides), fungi (fungicide), mites or ticks (miticides/acaricides), bacteria (bactericides), rodents (rodenticide), termites (termiticides), insects (insecticides), molluscs (molluscicides), and nematodes (nematicides), which form the basis of their classification (Anakwue, 2019). Another mode of pesticide classification can be based on their active ingredients, which include organochlorines, dichlorvos, diazinon, diamide, chlorpyrifos, etc. Although synthetic pesticides have positive effects on crop yield and productivity, they also have some negative impacts on soil biodiversity, animals, aquatic life, and humans (Farooq et al., 2019). Synthetic pesticides usually render the soil brittle, reduce soil respiration, and lessen the activities of some macroorganisms in the soil, such as earthworms (Pertile et al., 2020; Pelosi et al., 2021). They also reduce the characteristics of animal offspring, animal immunity to diseases, vitality, and the success of mating in animals (Syromyatnikov et al., 2020). They negatively affect soil microorganisms by limiting their biological services in the production of certain plant growth-promoting traits, such as siderophores, nitrogen, indole-3-acetic, etc. (Kumar and Kumar, 2019). When synthetic pesticides get into the environment through different means, such as vapormovements, indiscriminate disposal, droplet drift, erosion, and leaching, some non-targeted plants are encountered, thus resulting in a decline in the plant’s photosynthetic ability and seed production (Hashimi et al., 2020). The intrusion of pesticides into the water bodies during runoff can lead to the death of aquatic life and water pollution. Also, the accumulation of pesticides in the water bodies can be transitional from the aquatic lives to the animals and humans, and their biomagnification can result in deadly diseases, such as cancer, kidney diseases, skin rashes, diabetes, etc. (Jayaraj et al., 2017; Sabarwal et al., 2018; Manfo et al., 2020). However, biopesticides have emerged and have been very useful in the control of pests with lot of merits.

Biopesticides are cheap, environment-friendly, specific in their mode of action, sustainable, do not leave residues, and are not associated with the release of greenhouse gases (Borges et al., 2021). These biopesticides can be in the form of phytopesticides (plant origin; Idris et al., 2022), microbial pesticides (microbial origin; Harish et al., 2021), and nanobiopesticides (nanoparticles produced from biological agents; Abdollahdokht et al., 2022; Pan et al., 2023). Unlike synthetic pesticides, microbial pesticides are specific in action, can be easily sourced without the need for expensive chemicals, and are environmentally sustainable without residual effects (Harish et al., 2021; Hummadi et al., 2021). Phytopesticides have myriad of phytochemical compounds that make them exhibit various mechanisms of action, likewise, they are not associated with the release of greenhouse gases and are of lesser risks to human health compared to the available synthetic pesticides (Malahlela et al., 2021; Idris et al., 2022). Nanobiopesticides have higher pesticidal activity, targeted or controlled release with top-notch biocompatibility, and biodegradability compared to the synthetic pesticides (Abdollahdokht et al., 2022; Pan et al., 2023). Biopesticides act through different mechanisms, which include the inhibition and destruction of the plasma membrane and protein translation of pathogens/pests. Although, a few drawbacks have reduced their acceptability and commercial utilization, yet, biopesticides are highly specific in their target, have a short shelf life, are less persistent in the soil environment, and originate from sustainable raw materials, unlike synthetic pesticides (Kumar et al., 2021). Some of the merits of biopesticides mentioned above could also serve as their demerits. For example, the specificity in their target toward pest could be a demerit if the desire is to control many pests simultaneously. Also, their short shelf life means they are easily degradable and persist less in the environment, but this turns to a demerit if the goal is to completely eliminate the existing pests and prevent the growth of the pests that will come after the application of the biopesticides. The critical assessment of these merits and demerits, and the possible measures to improve on these seeming drawbacks has become very important. Therefore, this review examined the types, effects, advantages, and disadvantages of both synthetic and biopesticides. Also, different measures to improve on biopesticides (that is, microbial pesticides, phytopesticides, and nanobiopesticides) for possible incorporation into the integrated pest management system to reduce yield and quality loss were adequately discussed.

## 2. Classification of pesticides

**Figure 1:**
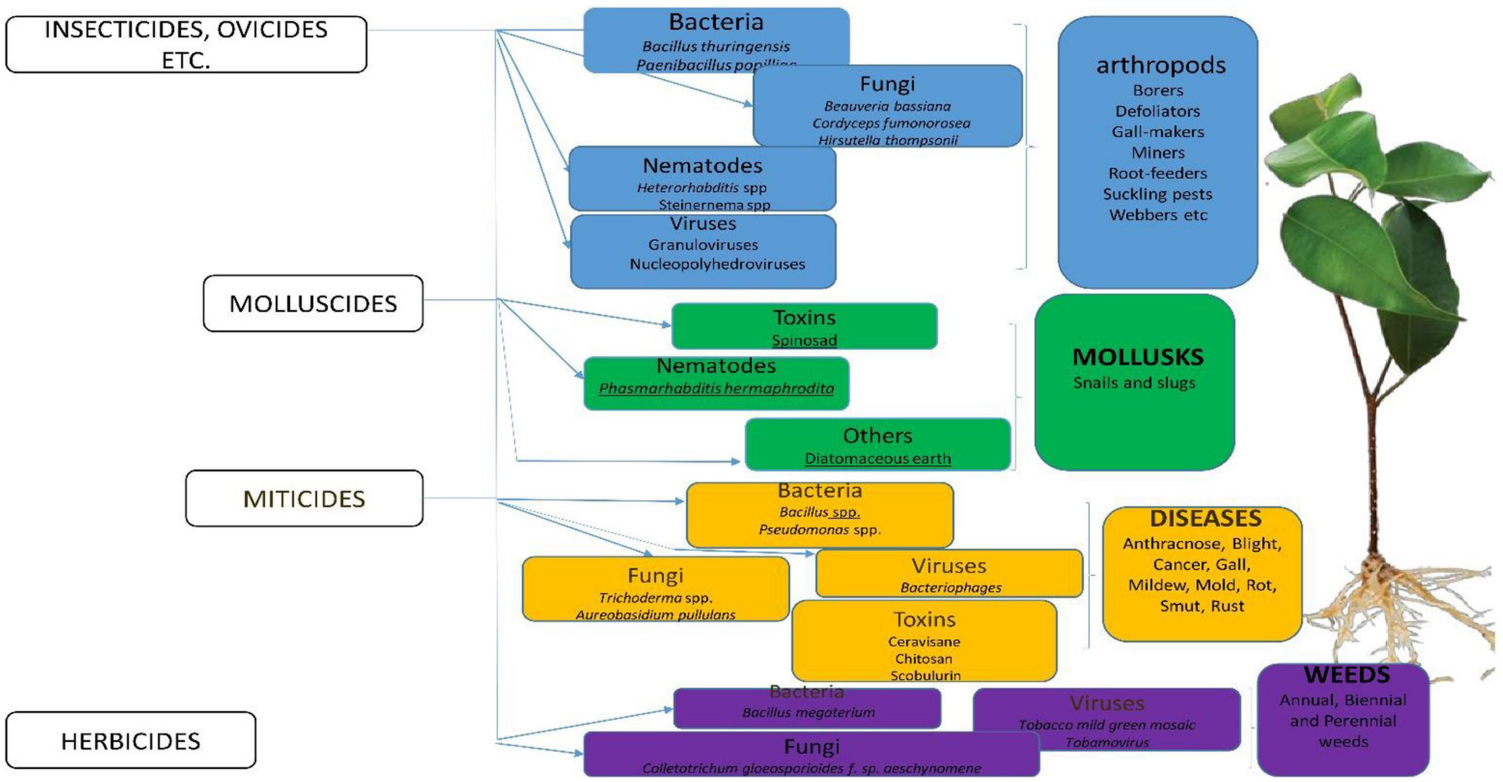
Classification of Bio-pesticides and their target pests.

Pesticides can be classified based on their active ingredients, functions, and sources. According to their active ingredients, pesticides are classified into organochlorines, propanil, and so on (Table 1). In terms of their functions, they can be classified into herbicides, fungicides, algicides, rodenticides, and so on (Figure 1). However, according to their sources, pesticides are classified into synthetic pesticides and biopesticides. Many pesticides, which include carbofuran, carbendazim, dichlorvos, anthraquinone, dinocap, paraquat, methomyl, aldicarb, and diuron have been banned for use in a lot of countries due to their toxicity to humans (Boucaud-Maitre et al., 2019). These banned pesticides are usually preferred by farmers because they are more affordable and more available compared to unbanned pesticides (Kehinde and Tijani, 2021). It is therefore important for World Health Organization (WHO), Food and Agriculture Organization (FAO), and other regulatory bodies to impose a ban on such products worldwide and also sanction companies that produce them.

**Table 1:** Classification of pesticides according to their active ingredients.

Biopesticides (Brand name)	Active ingredient	Pest controlled	Mode of action	References
Endosulfan	Organochlorines	Silkworm and Armyworm	It alters the enzymatic function and the electrophysiological properties of the nerve cell.	Anikwe et al. (2021); Indratin et al. (2021)
Mancozeb	Metal–organic compounds	*Phytophthora infestans*	It disrupts the biochemical processes within the cells of pests by interfering with the enzymes containing sulphydryl groups.	Sari and Lubis (2021)
Diazol	Diazinon	*B*. *invadens*	It inhibits the enzyme acetylcholinesterase (AChE), which hydrolyzes the neurotransmitter acetylcholine (ACh) in cholinergic synapses and neuromuscular junctions.	Abdullahi et al. (2020)
DDForce	Dichlorvos	*Phytophthora capsici*	It inhibits the neural acetylcholinesterase enzyme.	Aba et al. (2018); Okoroiwu and Iwara (2018)
Aldicarb	Carbamic and thiocarbamide derivatives	Thrips (*Frankliniella* sp.)	It inhibits the cholinesterase enzyme.	Allen et al. (2018)
Lefenuron	Urea derivatives	Dicotyledonous weed and broom corn plantation cereal	It interferes with the deposition synthesis, and polymerization, of chitin.	Abraham and Vasantha (2020); Ghelichpour et al. (2020); Surma et al. (2021)
Pyrinex Lorsban	Chlorpyrifos Chlorpyrifos	*B*. *invadens* Citrus peel miner larvae	It inhibits the cholinesterase enzyme.	Abdullahi et al. (2020); Maurer et al. (2018)
Sniper	Diamide	*S*. *exigua,* mosquitoes	It causes the misregulation of the ryanodine receptors (RyRs) in insects.	Ebuehi et al. (2017); Rabelo et al. (2020)
Cydim super	Cypermethrin+dimethoate	*B*. *invadens*	It modulates the sodium channel.	Abdullahi et al. (2020)
Talstar	Pyrethroid	*Agrotis ipsilon,* Tropical sob webworm	It modulates the sodium channel.	Rabelo et al. (2020); Campos et al. (2022)
				
Pendillin	Pendimenthalin	Weed	It inhibits root and shoots growth.	Dugje et al. (2020)
Laraforce	Lamdacyhalothrin	Insect	It disrupts gating by disrupting the gating mechanism of sodium channels.	Oso and Awe (2019)
Regalia	Chlorothalonil	Downy mildew and powdery mildew	It deactivates and reduces glutathione.	Jones et al. (2020); Scariot et al. (2022)
Deltapaz	Deltamethrin	*B*. *invadens*	It interferes with the normal production and conduction of nerve signals in the nervous system.	Abdullahi et al. (2020)
Alachlor	Chloroacetanilide	*B*. *stearothermophilus*	It blocks the synthesis of lipids and isoprenoids.	Pereira et al. (2021)
Roundup	Glycophosphate	Broomrape	It blocks the activity of the 5-enol-pyruvyl-shikimate-3-phosphate synthase (EPSPS) enzyme	Kanissery et al. (2019); Elsakhawy et al. (2020)
Dinocap	Phenol and nitrophenol derivatives	*Podosphaera pannosa*	It causes renal toxicity.	Kumar and Chandel (2018)
Caocobre	Copper-containing compounds	Cocoa black pod disease	It inhibits enzymes and disrupts the pest’s cellular proteins.	Husak (2015); Tsufac et al. (2020)
Paraeforce	Paraquat	Weed	It inhibits photosynthesis.	Imoloame (2021)
Aminforce	2,4-D Amine SL	Weed	It interferes with the hormones of the pest.	Dogara et al. (2022)
Butaforce	Butachlor	*Bufo regularis* tadpole	It inhibits cell division in pests.	Ejilibe et al. (2019)
Atrazine	Atrazine	Weed	It interferes with photosynthesis.	Sumekar et al. (2022)
Ronstar	Oxidiaxone	Weed	It inhibits the protoporhyrinogen oxidase (PPO) enzyme.	Dugje et al. (2020)

### 2.1. Classification of pesticides according to their functions

Pesticides can be classified according to the functions they perform. For instance, algicides (acaricides) destroy algae on different surfaces (Zheng et al., 2018), and antifeedants prevent the destruction of plants or harvested and preserved crops by other pests which could feed on them (Peprah-Yamoah et al., 2022). Herbicides prevent the growth of weeds and eliminate them (Loddo et al., 2019), while miticides are used to kill termites or ticks that destroy crops (Murcia-Morales et al., 2021). Similarly, bactericides and fungicides get rid of harmful bacteria and fungi, respectively, or inhibit their growth without tampering with the beneficial ones (Ullah and Dijkstra, 2019; Akanmu et al., 2021). Fumigants exhibit broad-spectrum activity against fungi, insects, and bacteria (Fang et al., 2020). Termiticides suppress the activities of termites on the soil (Singh et al., 2020). Repellents are used to repel insect pests and birds. Acaricides are used to control arachnids (Benelli, 2022) and insecticides help to destroy insects that affect plants, animals, and humans (Matsuda et al., 2020). Equally, the effective use of nematicides in the control of nematodes, rodenticides in the control of mice and other rodents, and molluscicides in the control of molluscs have been documented (Horgan and Kudavidanage, 2020; Figure 1). Attractants lure and attract pests to a trap or bait (Souto et al., 2021). Insect growth regulators disrupt the molting, maturity from pupal stage to adult, or other life processes of insects (Jindra and Bittova, 2020).

### 2.2. Classification of pesticides according to their sources

Pesticides are classified into chemical and biological pesticides according to their source. Chemical pesticides are very effective and rapid in the control of pests. They are made from inorganic or synthetic salts, such as sulfur, copper sulfate, lime, and ferrous sulfate. Their chemical compositions are simple and highly soluble in water, which makes them easily absorbable by pests, thereby enhancing their activity and durability in the environment (Kim et al., 2017; Abubakar et al., 2020).

Biological pesticides (biopesticides) are substances produced from biological agents that manage pests in agriculture to enhance crop production (Samada and Tambunan, 2020). They can be sourced from microorganisms, plants, or nanoparticles (Kidd et al., 2017; Samada and Tambunan, 2020; Adeleke et al., 2022d). Microbes release certain metabolites, which protect plants from pests and are useful as microbial pesticides (Samada and Tambunan, 2020). Active compounds from plants used as phytopesticides include phenols, alkaloids, and terpenes (Abubakar et al., 2020). Generally, nanoparticles can be produced from chemical or biological agents (mainly plants or microbes; Omole et al., 2018). Nanoparticles of biological origins that are used as pesticides are termed nanobiopesticides and are also very important as plant protectants (Pan et al., 2023). Nanobiopesticides have found application as pesticidal agents in agriculture because of their excellent physicochemical characteristics like size, reactivity, surface area, and so on. Besides, nanobiopesticides have unambiguous biological interactions with plants, as well as clear transport and fate in the environment (Bratovcic et al., 2021; Kumar et al., 2022; Pan et al., 2023).

### 2.3. Adverse effects of synthetic pesticides

Synthetic pesticides are faced with drawbacks, which include the cost of purchase and production, persistence in the soil, pest resistance, impacts on health and the environment, economic harm to organic producers due to pesticide drift, disposal of contaminated crops, removal of stockpiles of unused pesticides as well as regular containers, and the disposal of expired/unused products, which can affect organic farms or innocent populace (Hicks et al., 2018; Essiedu et al., 2020).

A large portion of pesticides when applied on the soil for agricultural purposes remains non-degradable. Hence, they are more persistent in the environment and leach to underground and surface water, thus leading to loss of biodiversity and pollution. Of all the pesticides applied on the soil, about 98% affect organisms that are not targeted. For instance, in Europe, pesticides decrease soil respiration by 35%, reduce insect biomass by 70%, and decrease the number of farm birds by 50%; and in America and Europe, it reduces the honeybee population by 30% (Ali et al., 2021). Research by Tongo et al. (2022) revealed Aldrin pesticide as a major pesticide detected in the Ikpoba river in the Southern part of Nigeria. Although, other chemicals, such as diazinon, endrin, glyphosate, aldrin, endosulfan I, heptachlor, heptachlor epoxide, and carbofuran present with the tendencies of being biomagnified need proper monitoring. Furthermore, pesticides (e.g., carbamate and organophosphate) have been reported to negatively affect soil’s nutrients, as they chelate some important metal ions, thus making them unavailable for plant uptake (Kaur et al., 2017). Likewise, plant photosynthesis, reproduction, and seed production can be adversely affected by pesticides (Hashimi et al., 2020).

Residues of pesticides in food crops can be consumed directly by humans or used in the production of animal feeds (Choudhary et al., 2018). This can come to play when pesticides are applied toward harvesting (Jallow et al., 2017). Biomagnification of pesticides occurs in animals when they feed on accidentally or deliberately contaminated harvested food crops or forage. Topical pesticides are applied on food crops to control parasites, and through other means, such as disposal, spraying, and formulations of pesticides (Choudhary et al., 2018). Pesticide accumulation in the granular tissues of animals can lead to the death of cells, necrosis (causing a reduced hormone production), ovarian follicles (resulting in a reduced progesterone level), reduced oestrogen production, reduced libido, and a reduced sperm concentration and quality in male animals (Li et al., 2022).

Accumulation of pesticides in birds (e.g., bald eagles, ospreys, grebes, cormorants, seagulls, pelicans, and peregrine falcons) living in pesticide-polluted areas can lead to reproduction problems (Garces et al., 2020). Pesticides lead to crossed bill deformity in birds. For example, a high concentration of DDT pesticides led to crossed-bill deformity in a wild bald eagle (Garces et al., 2020). In reptiles inhabiting areas close to rivers where water from agricultural farms is washed, deformities could be observed. For example, snapping turtles living in Erie and Lake Ontario in Canada were found to have deformities, such as deformed jaws, limbs, cranium, carapaces, nostrils, and tails, enlarged yolk sacs, dwarfism, missing eyes, unhatched eggs, and these were traced to chemical pesticides contamination (Garces et al., 2020). In soils, the reduction in the function and population of fungi, actinomycetes, and bacteria has been linked to the usage of three pesticides, namely glyphosate, malathion, and alphacypermethrin (Kumar et al., 2019). All the negative effects of synthetic pesticides lead to the loss of biodiversity and genetic conservation in animals. Furthermore, it also alters soil biodiversity and health, by affecting the microbial functions in the soil, which directly or indirectly enhances soil nutrients and plant health.

Consumption of vegetables, food crops, fruits, milk, and meat from animals that contain high pesticide residue can lead to different diseases in humans (Omoyajowo et al., 2018; Li et al., 2022). Onwujiogu et al. (2022) found pesticides in Bambara groundnut quantity, which is beyond the Maximum Residual Limit (MRL) recommended by the WHO and could pose a threat to the health of humans, especially children who feed on them. Omoyajowo et al. (2018) also experimented to unravel the level of pesticides in three fruits and realized that the pesticide level of watermelon was above the MRL level specified by the WHO/FAO, which equally poses a health threat to the consumers. Similarly, pesticides are used to protect harvested food crops, vegetables, and fruits and those used for other purposes aside that which they are manufactured. For instance, the use of calcium carbide to ripen fruits poses health threats to humans. Calcium carbide contains calcium arsenide and calcium phosphide, and when reacts with water, forms arsine and phosphide, thus leading to headache, vomiting, dizziness, nausea, unconsciousness, and fatigue in humans (Andrew et al., 2018). Equally, ethepon, a pesticide used to hasten the ripening of fruits, vegetables, and cereals exhibited hepatocyte properties when tested on albino rats (Bhadoria et al., 2018).

Furthermore, in humans, biomagnification of pesticides through food (such as fish), drinking water, skin pores (while spraying), post-harvest crop preservation, and inhalation, give rise to diseases, such as cancer, Parkinson’s diseases, eye irritation, diabetes, kidney diseases, hypertension, skin rashes, liver dysfunction, eczema, birth defects, Alzheimer’s diseases, neurological destruction, cardiovascular diseases, and endocrine disorder (Damalas and Koutroubas, 2016; Jayaraj et al., 2017; Sabarwal et al., 2018; Manfo et al., 2020). Likewise, high pesticides level can lead to about 25–30% increase in mental ailments, and a 50% increase in severe brain cancer, leukaemia, lymphoma, and cancer.

## 3. Biopesticides as an alternative to synthetic pesticides

**Figure 2:**
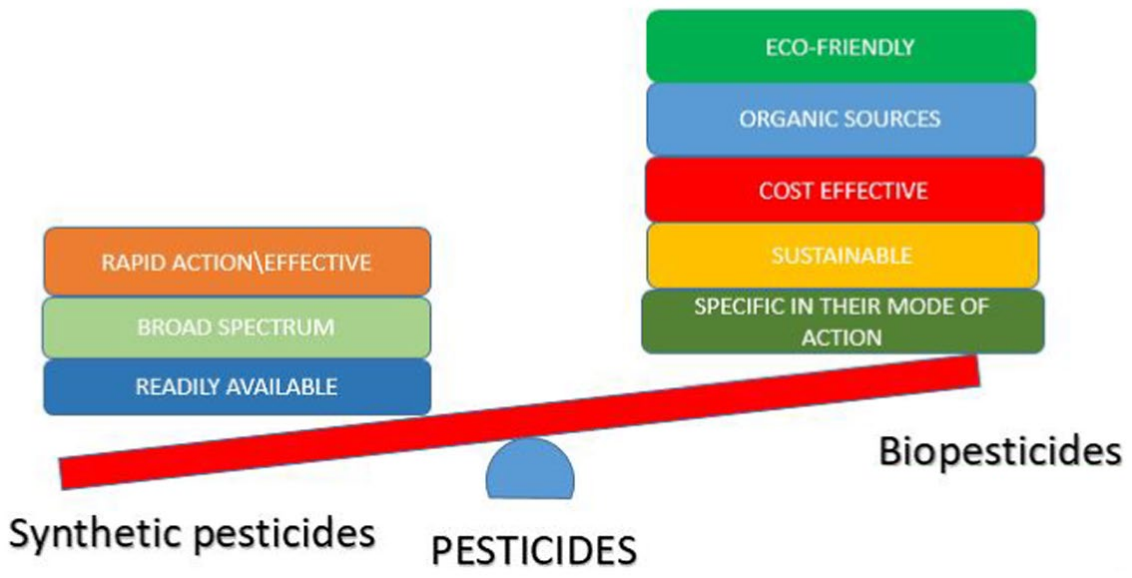
Comparison of the advantages of synthetic pesticides and biopesticides.

Due to the drawbacks of synthetic pesticides, an alternative means of pest control is being encouraged, which is the use of biopesticides (Ojuederie et al., 2021; Ayilara et al., 2022b; Figure 2). Biopesticides are effective and safer means of controlling pests, they have a mild effect on the environment compared to their synthetic counterpart, and they are specific in their target, hence preventing bioaccumulation (Saberi et al., 2020; Kumar et al., 2021). Biopesticides are made from natural substances, such as plants, microbes, and nanoparticles of biological origin, thus, making them a sustainable means of pest control (Kumar et al., 2021).

Some successes have been recorded in the use of biopesticides in the control of some pests to which chemical pesticides are being applied (Table 2).

**Table 2:** Different Biopesticides and the pest they control.

Microbial pesticides			
	Entomopathogenic viruses	Target insects and pests	References
	Nucleopolyhedroviruses	*Lepidoptera*	Harish et al. (2021)
	Imported cabbageworm (PiraGV) NPV (AucaMNPV)	*Artogeia (Pieris) rapae*	Singh et al. (2019)
	Potato tuber moth GV (PhopGV)	Phthorimaea operculella	Singh et al. (2019)
	**Entomopathogenic fungi**		
	*Paecilomyces lilacinus*	Soil nematodes	Moreno-Gavíra et al. (2020)
	*Beauveria bassiana*	Whitefly	McGuire and Northfield (2020)
	*Hirsutella thompsonii*	Spider mites and whitefly	Saranya et al. (2021)
	*Isaria fumosorosea*	Termites, grasshoppers, caterpillars, and beetles	Gautam (2020)
	*Metarhizium brunneum*	Nematodes (pathogens)	Hummadi et al. (2021)
	*Paecilomyces fumosoroseus*	Insects and mealy bugs	Abbas (2020)
	*Verticillium lecanii*	Nematodes, mites & thrips, scale insects, mealy bugs, etc.	Pathania et al. (2022)
	*Myrothecium verrucaria*	Nematodes	Hagag (2021)
	*Lagenidium giganteum*	Pest mosquito species	Kaczmarek and Boguś (2021)
	**Entomopathogenic bacteria**		
	*B*. *thuringiensis*	Elm Leaf Beetle,Alfalfa weevil	Saberi et al. (2020)
	*Beauveria bassiana*	Whitefly	McGuire and Northfield (2020)
	*B*. *thuringensis* var. *israelensis*	Fungus gnats, black flies, larvae of mosquitoes	Lee et al. (2021)
	*B*. *sphaericus*, *B*. *lentimorbus*, and *B*. *popilliae*	Larvae of Aedes spp., Culiseta, Psorophora, and Culex mosquitoes	Falqueto et al. (2021)
	**Entomopathogenic nematodes**		
	*Heterorhabdits taysearae*	*Bactrocera dorsalis*	Godjo et al. (2018)
	*Steinernema carpocapsae, Heterorhabditis bacteriophora,*	Larvae of cabbage white butterfly	Aioub et al. (2021)
	*Steinernema feltiae*		
	*Steinernema carpocapsa,*		
	*Steinernema riobrave,*	Armyworm	Gozel and Gozel (2021)
	*Steinernema feltiae*		
	*Steinernema carpocapsae,*		
	*S*. *bicornutum,*	Leafminers	Abbas (2022)
	*Heterorhabditis indica* and		
	*H*. *bacteriophora*.		
	*Steinernema carpocapsae*		
		Potato tuber moth	Ebrahimi et al. (2022)
**Nanobiopesticides**			
	**Nano-sized particles**		
	*Mesocyclops longisetus*-derived nanoparticles	*Culex quinquefasciatus*	Narware et al. (2019)
	*Mesocyclops scalpelliformis*-derived nanoparticles	*Culex quinquefasciatus*	Rodrigues et al. (2019)
	Silver nanobiopesticide	*Alternaria solani, A*. *alternata*	Narware et al. (2019)
	Silver nanoparticles	*Xanthomonas axonopodis pv*. *citri, X*. *oryzae pv*. *oryzae* and *Ustilaginoidea virens*	Roseline et al. (2019)
	Gold nanoparticles	*Culex quinquefasciatus, Anopheles stephensi* and *Aedes aegypti*	
			Kovendan et al. (2018)
**Phytopesticides**			
	**Plants**		
	*Lantana camara*	Eggs of root-knot nematode- *Meloidogyne incognita*	Malahlela et al. (2021)
	*Azadirachta indica*	*Colletotrichum coccodes*	Opeyemi et al. (2018)
	*Jimson weed, Camelina* and *White hellebore*	*Colorado beetle*	Basiev et al. (2019)
	*Andropogon nardus*	*S*. *rolfsii and Pestalotia sp*	
	*Atalantia guillauminii, Eucalyptus procera*	*Tenebrionid pests*	Idris et al. (2022)
	*Siparuna guianensis*	*Lepidoptera sp*.	
			Lourenço et al. (2018)

**Figure 3:**
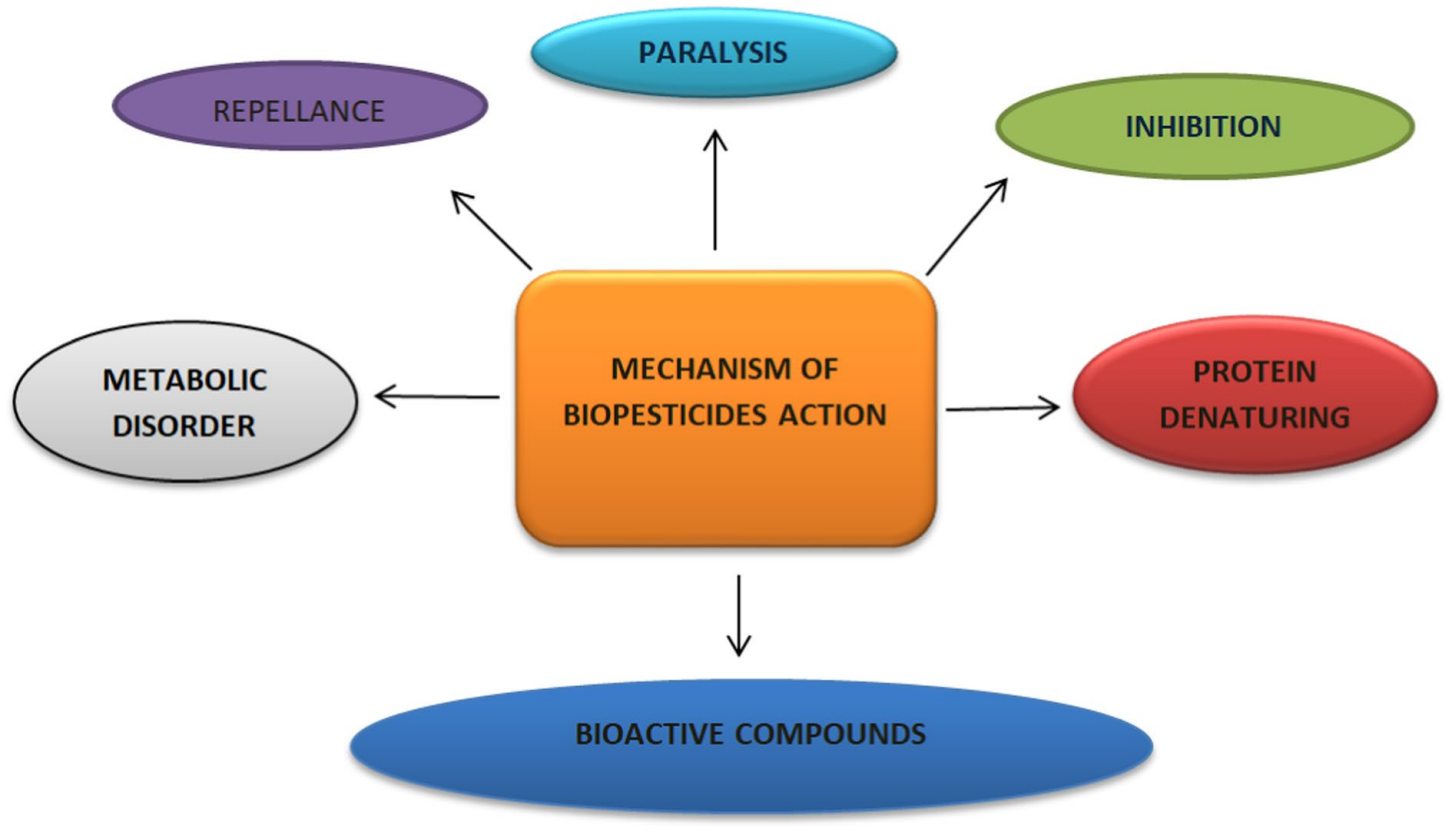
Mechanism of action of biopesticides.

The effectiveness of biopesticides in pest management comes from various modes of action, which include actions that regulate gut disruption, pest growth, and pest metabolism. Biopesticides work by denaturing protein, causing metabolic disorder and paralysis, activating target-poisoning mechanisms, exhibiting multisite inhibitory actions, and releasing neuromuscular toxins and bioactive compounds (Figure 3; Sparks and Nauen, 2015; Dar et al., 2021; Fenibo et al., 2021). These multiple actions offer biopesticides the capacity to alter the course of pest resistance as compared to chemical pesticides. Studies have indicated that biopesticides are eco-friendly, possess low toxicity properties, are biodegradable, and specific in action with little or no negative impact on non-target organisms (Deravel et al., 2014; Kalpana and Anil, 2021), Unlike biopesticides, conventional pesticides are a major source of environmental pollution, which promotes pest resistance with high post-harvest contamination and bioaccumulation in food crops (Fenibo et al., 2021).

However, there are various limitations to the full adoption, development, and use of biopesticides in agriculture. Biopesticides are often ranked as having low efficacy and a slower rate in the control of pests and diseases (Damalas and Koutroubas, 2016; Delgado‐Carrillo et al., 2018). Commercial biopesticide products are highly expensive and not readily available in the global market. In addition to the problem of commercialization, biopesticides also face quality control problems and concise shelf-life (Arthurs and Dara, 2019).

Many farmers also worry about dosage recommendations and fear the evaluation of new pest species that may be resistant to the existing biopesticides (Stevenson et al., 2017). The advantages and disadvantages of biopesticides are summarized in Table 3. Biopesticides have also been classified into three groups based on their extraction source and the constituting molecule/compound. The three groups include; (i) Microbial Biopesticides; (ii) Biochemical Pesticides; and (iii) GMO-Based Biopesticides. The source characteristics and the consisting molecules of biopesticide influence the mechanisms by which biopesticides protect the crops from the attack of pathogens. For example, fungicides and bactericides derived from microorganisms act by inhibiting or disrupting the process of protein translation, or cause a major disruption in plasma membrane permeability, thus leading to cell death, while some may prevent glucose formation in target pathogens (Parker and Sperandio, 2009; Svidritskiy, et al., 2013; Gwinn, 2018).

**Table 3:** Advantages and disadvantages of biopesticides.

Pesticides	Advantages	Disadvantages	References
Microbial pesticides	They are specific in their mode of action, have a short residual effect, they are environment friendly, are made from different species, which ensures sustainability in their production, are cost-effective, and it is easy to make a mass production *in vitro*. They are not associated with the emission of greenhouse gases.	They have short shelf lives, there is a challenge with their stability in different environments, and there are uncertainties regarding the exposure rate/level and duration. They are easily degraded and their effects last for a short period. In many countries, the regulations for their registration are very stringent which reduces their availability.	Borges et al. (2021); Kumar et al. (2021); Llamas et al. (2021); Adeleke et al. (2022c)
Plant pesticides	They are cost-effective and sustainable compared to synthetic alternatives. They can be derived from different plant species.	They are specific; thus may not be able to control more than one pest at a time. Therefore, any plant that has the potential to be affected by more than one pest may require more than one phytopesticide. Their quality is dependent on the quality of the raw materials used; therefore, the plant materials used must be harvested during the time of the day when the plant phytochemicals are active. There might be issues with the consistency of products because the concentration and constituents of plant phytochemicals change across different geographical and ecological locations. Their registration and registration procedure are tedious.	Damalas and Koutroubas (2020); Souto et al. (2021)
Nanobiopesticides	They are cheaper, more stable, and sustainable compared to their chemical counterparts; they have no residual effects and are not associated with greenhouse gases emission.	The dosage of nanoparticles in the environment might be difficult to control because they are small in size and because many nanoparticles, which are occurring naturally might be identical to the ones introduced to the environment. They are more active in the laboratory, and not much work has been done on their field application. The procedure for their regulations is tedious and time-consuming and they have a slow mode of action.	Chaudhary and Sharma (2019); Damalas and Koutroubas (2020); Sabry (2020); Saleh (2020); Adeleke et al. (2022a)

### 3.1. Microbial pesticides

Microbial pesticides consist of substances derived from microorganisms like bacteria, fungi, viruses, protozoa, and algae, which are used in the control of pests (Adeleke et al., 2022c). Microbes use the toxic metabolites produced to destroy and prevent the growth of pests. Microbial pesticides are applied to the environment through different techniques, such as emulsion, electrospraying system, fluidized bed, spray drying, extrusion, lyophilization, spray cooling, and coacervation (De Oliveira et al., 2021). The major categories of microbes used as biopesticides include bacteria genera, *Chromobacterium, Pseudomonas,* and *Yersinia*, fungal genera *Beauveria, Paecilomyces, Verticillium, Hirsutella, Metarhizium*, and *Lecanicillium* and nematodes belonging to the genera *Steinernema* and *Heterorhabditis* (Chang et al., 2003; Kumar et al., 2021; Adeleke et al., 2022b).

A fungi species, *Trichoderma* sp. has been reported to prevent the activity of numerous fungi inhabiting the soil that cause root rot, black gram, and green gram in chickpeas, and groundnut (Samada and Tambunan, 2020). Likewise, *Beauveria bassiana* and *M*. *brunneum* have been reported in the control of thrips, beetles, weevil, aphids, whiteflies, and mites infestation in ornamental crops, fruits, and vegetables (Dara, 2017; Arthurs and Dara, 2019). Other examples of microbial pesticides are listed in Table 2.

Of all the bacterial pesticides*, Bacillus thuringiensis* (Bt) is well-known and have been made into products available for commercial purpose (Ujvary, 2010; Ruiu, 2018). *Bacillus thuringiensis* is a Gram-positive bacteria that acts as an insecticide by producing exudates, such as poisonous parasporal crystals and endospores which when consumed by insects get dissolved in their midgut by the alkaline environment and release delta-endotoxin, a protein that has a lethal effect on insects (Xiao and Wu, 2019). *Bacillus* thuringiensis is used to reduce pest infestation in plants, such as cabbage and potato, and is capable of controlling lepidopterans in different plants (Berini et al., 2018; Samada and Tambunan, 2020). As good as the positive effects of commercially available Bt sounds, they come with some drawbacks which include quick deactivation when exposed to light, short activity time, slow lethal rate, and low awareness and sensitivity to the environment (Xiao and Wu, 2019). The short life and environmental sensitivity of microbial pesticides, which reduce their awareness and usage are the major challenges associated with their use (Pathak et al., 2017). For instance, baculoviruses only survive in their host and cannot reproduce outside their host; hence, they cannot be used outside their host (Borges et al., 2021). Their host may have an adverse environmental impact on the environment, and their use might be dangerous.

Fungi are also used to control plant pests. An example is the mycoinsecticide, which is a microbial insecticide whose active ingredient is a living fungus that exhibits an antagonistic effect on insects or other arthropod pests, with some strains releasing metabolites while inside the pest that may also injure or kill it (Zaki et al., 2020). Only a few rare fungal strains have been developed as commercial mycoinsecticides, hence, the technology is still in its early stages. Attachments, germination, penetration, invasion, replication, and host death are the six general phases of action for mycoinsecticides (Zaki et al., 2020).

Spores can land on and attach to the target host’s cuticle when the formulated product is diluted and applied according to label instructions. Adhesion is primarily achieved through hydrophobic interactions between the cuticle and the spore. The number of spores attached to the host’s body determines their efficacy. The spore germinates in response to chemical cues on the cuticle and then develops an aspersorium, which is the penetration structure. The fungus penetrates the layers of the cuticle through a combination of mechanical pressure and enzyme degradation (Zaki et al., 2020).

Generally, microbial pesticides exert no adverse effects on the environment, producers, and consumers of agricultural products because their ingredients are generally considered safe and are target-specific (Guven et al., 2021). In addition, their usage lower greenhouse gas emissions compared to chemical pesticides (Llamas et al., 2021). Furthermore, there is a wide variety of organisms from which microbial biopesticides can be derived to solve the problem of resistance and ensures sustainability. Since different microbes used as biopesticides might require different storage condition, it might be cumbersome for sellers, producers, marketers, and end users to cope with their storage and transportation. Hence, more research is needed to ensure a sustainable and extended shelf-ability of microbial pesticides.

### 3.2. Phytopesticides

Essential oil and extract from different parts of plants have been successfully used to control plant diseases (Ayilara et al., 2022a). They attract, repel, prevent respiration, detect host plants from specific pests, destroy the eggs and larvae of pests, and destroy pests from feeding on plants (Tripathi et al., 2009; Halder et al., 2013; Ali et al., 2017). Essential oil from *Coleus aromaticus* Benth., *Hyptis suaveolens* (L.), *Azadirachta indica, Ageratum conyzoides* L., and *Achillea* sp., have been reported to control the infestation of *Tribolium castaneum* (Herbst), a red flour beetle that destroys many crop species (Singh et al., 2014; Jaleel et al., 2015; Upadhyay et al., 2018). Other plant parts, such as bark, flowers, roots, leaves, peels, seeds, and buds can be used to control different plant pathogens (Tongnuanchan and Benjakul, 2014).

Plant families that have been reported to contain bioactive compounds with activity against important crop pests include Myrtaceae, Lauraceae, Rutaceae, Lamiaceae, Asteraceae, Apiaceae, Cupressaceae, Poaceae, Zingiberaceae, Piperaceae, Liliaceae, Apocynaceae, Solanaceae, Caesalpinaceae, and Sapotaceae (Gakuubi et al., 2016). They are easily available which makes them inexpensive and can be easily incorporated into agricultural production systems. Secondary metabolites, such as steroids, alkaloids, tannins, terpenes, phenols, flavonoids, and resins are commonly found in botanical pesticides and have shown antifungal, antibacterial, antioxidant, or insecticidal properties (Ahmad et al., 2017). The specific compounds found in certain plant species make them effective against a specific category of pests and also determine their mode of action on the target pests (Lengai et al., 2020). Botanical pesticides contain bioactive compounds that act in a variety of ways against pests, such as insects, fungi, bacteria, nematodes, and plant host cells infected with viral pathogens (Lengai et al., 2020). Depending on the botanical compound and pest, the modes of action may include repellence, inhibition, protein denaturation, and other effects. Pesticides derived from pyrethrum target insect nerve cells, thus causing paralysis and death. Also, neem-based pesticides with antifeedant and repellent properties, induce moulting abnormalities, hinder oviposition, and disrupt the endocrine system (Lengai et al., 2020).

Pesticides from plants have been well-reported to interfere with the normal metabolism of insect pests, which include the octopamine and acetylcholinesterase pathways (Polsinelli et al., 2010; Pang et al., 2012; Dassanayake et al., 2021). Acetylcholinesterase is an enzyme used by insects in their neuronal communication and neuromuscular functions and can be toxic to insects by destroying the membrane of the postsynaptic junction and the current of the nerve. Octapamine on the other hand is a hormone involved in neuromodulation and neurotransmission in insects and can impair the muscle juncture and homeostasis of the body fluids of insects (Dassanayake et al., 2021). Equally, plant pesticides can prevent cell wall biosynthesis, cell membrane structure, ATPases function, quorum sensing, efflux pumps, and biofilm formation (Lang and Buchbauer, 2012; Hu et al., 2017). Extracts from four weed plants, namely *Lippia javanica*, *Tithonia diversifolia*, *Vernonia amygdalina,* and *ephrosia vogelii*, in Tanzania were used to control insects in common bean (Mkenda et al., 2015). Similarly, Lovatto et al. (2004) carried out an experiment where nine different aqueous plant extracts from the leaves, fruits, and flowers of nine plants were used to repel and kill *Brevicoryne brassicae*. *Solanum pseudocapsicum* L., and *Solanum guaraniticum* A were reported to be the most effective.

### 3.3. Nanobiopesticides

Nanobiopesticides can be defined as biological protection products that are developed using nanotechnology to enhance efficacy and reduce an environmental load of pesticides (Chaudhary et al., 2021b,d). Nanobiopesticides are formulated from nanomaterials and applied specially fixed on a hybrid substrate, encapsulated in a matrix or functionalized nanocarriers for external stimuli or enzyme-mediated triggers (Agostini et al., 2012; Khati et al., 2018; Kumari et al., 2020; Agri et al., 2021, 2022; Chaudhary et al., 2022; Pan et al., 2023). They are nanostructures with two or three dimensions used for carrying agrochemical ingredients and can help increase water solubility and bioavailability, and protect agrochemicals against environmental degradation. It also helps revolutionize the control of pathogens, weeds, and insects in crops (Yadav et al., 2020). They are available in different forms, such as nano-gel, nano-encapsulation, nano-fibres, nano-sphere, etc. (Rajna and Paschapur, 2019; Pan et al., 2023).

Nanoparticles in recent years are being reported to be very helpful in agriculture (Omole et al., 2018). They have been used as active ingredients and carriers to stabilize many agrochemicals and their products from them include nanofertilizers, nanopesticides, etc. (Chaudhary and Sharma, 2019; Chaudhary et al., 2021a). For instance, pesticides from nanomaterials, such as magnesium oxide, magnesium hydroxide, copper oxide, and zinc oxide derived from aqueous extracts of *Chamaemelum nobile* flowers, *Punica granatum* peels, green peach aphid (GPA) and *Olea europaea* leaves have been reported in the control of insects (Grillo et al., 2021; Konappa et al., 2021). Also, silver nanoparticles derived from the leaf extract of *Euphorbia hirta* have been explored in the control of the causative agent of cotton bollworm, *Helicoverpa armigera* (Devi et al., 2014). The ability of copper oxide nanoparticles and zinc oxide nanoparticles to control *Alternaria citri*, a causative agent of citrus black rot disease in the plant has as well been reported (Lasso-Robledo et al., 2022). In addition, Sardar et al. (2022) used combined and individual zinc oxide and copper oxide to control citrus black rot disease in a potato dextrose medium. The fungal and insecticidal effects of copper nanoparticles have been demonstrated against *Tribolium castaneum,* a pest that affects grain (El-Saadony et al., 2020). The major interactions which occur between plants and nanoparticles have been studied using different techniques, which include fluorescence spectroscopy, microscopy, and magnetic resonance imaging (Chhipa, 2019). The effectiveness of nanobiopesticides can be determined by the composition, surface charge, concentration, size, and chemical and physical changes (Chhipa, 2019).

The critical role of nanoformulations in reducing active ingredient degradation, improving water solubility equilibrium, and increasing the biological availability of active ingredients is well understood, and this has helped in avoiding endemic pest infestation, plant injury, and economic loss by lowering the quality and quantity of agricultural products and foods (Syafrudin et al., 2021; Chaudhary et al., 2021a,c).

Because of their small size and larger surface area, nanopesticides’ chemical properties differ significantly from conventional pesticides, and these properties can be used to develop an efficient assemble of a structure with several advantages, such as the possibility of better interaction and mode of action at a target site of the desired pest. Nano-sized products exhibit greater selectivity without impairing compound bioactivity against the target pathogen. Their increased toxicity can also increase pest penetration (Priya et al., 2018). Nanoparticle application reduces drifting and leaching issues and allows for the use of a smaller amount of active compound per area, as long as the formulation can provide optimal concentration delivery for the target insecticide for longer periods. There are several methods for creating pesticide nanoproducts, such as nanoemulsions, nanocapsules, and inorganic engineered nanoparticles (such as metal oxides, metals, and clays), and can be further developed to improve the efficacy of existing pesticides, reduce their environmental toxicity, or both.

On a general note, biopesticides have been reported to be capable of controlling pests but their sole use for sustainable agriculture may not be realistic, majorly because they are not readily available in many locations and their mode of action can be very slow. Hence, they should be incorporated with the existing synthetic pesticides and be applied majorly close to the harvest period of crops since residual chemicals observed in plants are those majorly applied close to harvest periods. Furthermore, this will help to maintain suitable agriculture, pending the improvements of biopesticides.

### 3.4. Molecular mechanisms of the application of biopesticides

It is very important to understand the molecular mechanisms underlying the action of biopesticides at each stage of action to ensure better control strategies over pests. Understanding the biopesticides mechanisms of action against insect pests at the molecular level will allow for synergistic approaches among biopesticides, which have different mechanisms of action without an overlapping mechanism. This will also give allowance for the exploration of different toxic molecules present in biopesticides that can enlarge the pesticidal arsenal of these biopesticides. The widely used biopesticides and their mechanisms of action at the biochemical level have been described. However, the entomopathogenic fungus, *Beauveria bassiana* has gained wide acceptance and can be used as a model to describe the molecular mechanism of biopesticides’ application.

*Beauveria bassiana* is an example of an entomopathogenic fungus that has been widely used as biopesticide because it is highly efficacious against a lot of arthropod hosts (Boomsma et al., 2014). However, to understand their effectiveness and sustainability against pests, there is a need to fully evaluate their molecular mechanism of pathogenicity beyond the conventional approach. The mechanism of pathogenicity of *B*. *bassiana* begins with adhesion to the host pest, penetration of cuticle, and colonization of the pest heaemocoel (Wojda et al., 2009).

The hydrophobins-coated aerial conidia of *B*. *bassiana* allow its hydrophobic interaction with the cuticles of insects (Holder and Keyhani, 2005). This hydrophobicity of the *B*. *bassiana* aerial conidia can be influenced by the role that several genes expressed by *B*. *bassiana* play in lipid homeostasis. It has been revealed by transcriptomics analyses that there is an upregulation of gene expressions for hydrophobins and *Metarhizium* adhesion-like protein 1, 2 (MAD 1, MAD2) by *B*. *bassiana* which are crucial for its hydrophobic attachment to the cuticle of insect (Wang and St Leger, 2007). The transportation and storage of lipids in the conidia, and maintenance of the lipid homeostasis of *B*. *bassiana* is possible when mammalian-like perilipin 1 (MPL1) genes are over-expressed (Chen et al., 2018). The role that the MPL1 gene plays is crucial because its deletion causes a reduction in the turgor pressure of the appressoria impairing the adhesiveness of *B*. *bassiana* (Wang and Leger, 2007). Also, the surface sensing and signaling for the germination of conidia and formation of appressoria is made possible by CFEM-domain-containing genes in *B*. *bassiana* (Sabnam and Barman, 2017). Proteomics has also revealed that *B*. *bassiana* secretes sphingomyelin phosphodiesterase, which allows it to disrupt the membrane of the host insect upon contact with the cuticles of the insect (Santi et al., 2019).

Once *B*. *bassiana* completed adhesion to the host insect, its conidia germinate and develop appressoria to allow penetration into the cuticle of the host. The penetration efficiency of *B*. *bassiana* usually increased when the structural outlook of the appressorium allows the synergistic functioning of enzymatic digestion and mechanical pressure (Singh et al., 2017). The hyphae of *B*. *bassiana* germinate in the exoskeleton of the insect as the penetration proceeds and *B*. *bassiana* produces secondary hyphae inside the cuticle. The hyphae switch to blastospores (motile, more hydrophilic, and better evade the insect’s host immunity) when exposed to hyperosmotic environment in the haemocoel (Ortiz-Urquiza and Keyhani, 2016). Through transcriptomics, it has been reported that chitin synthase is responsible for chitin production, and β-1,3-glucanases soften the cell wall to allow germination, while several cell wall protein-conferring genes give the cell wall of *B*. *bassiana* its building blocks (Tartar et al., 2005; Mouyna et al., 2013; Chen et al., 2018). Genes necessary for the cell body differentiation in *B*. *bassiana* include osmosensor Mos1, signaling-related genes, and mitogen-activated-protein kinases (MAPKs) like protein kinase A (PKA; Chen et al., 2018; Zhou et al., 2019). For penetration into the cuticle of the host insect, notable proteases, lipases, chitinases, and carboxypeptidases have been reported and these include subtilisin-like protease (Pr) isoform 1A (Pr1A) and 1B (Pr1B), cytochrome P450s (CYPs) and GH18 family chitinases (Lai et al., 2017).

In response to the penetration into the cuticle of the insect, the insect activates melanization and produces antimicrobial peptides (AMPs), reactive oxygen species (ROS), and protease inhibitors (Ortiz-Urquiza and Keyhani, 2016). Stress management and immune-evasion-related genes are upregulated to overcome the host insect defense mechanisms. Glutathione S-transferases (GSTs), catalases, peroxidases, superoxide dismutase (SODs), thioredoxins, and oxidoreductases are anti-oxidative enzyme-producing genes over-expressed in *B*. *bassiana* (Lai et al., 2017). Heat shock proteins (HSPs) are expressed to maintain internal cellular integrity against diverse types of stress (Santi et al., 2019). Another mechanism used by *B*. *bassiana* is the production of secondary metabolites that are toxic to the insect cell. These metabolites include oosporeins, beauvericin, isarolides, beauverolides, tenellins, and bassianolide (Chandler, 2017). The biosynthesis of oosporein happens in the haemocoel and it is mediated by the over-expression of polyketide synthase (PKS) gene (Lai et al., 2017). It is interesting to note that a greater amount of beauverolides secreted by *B*. *bassiana* usually occur when live insect tissues are present than in the presence of dead insect tissues (de Bekker et al., 2013). With these fantastic mechanisms of action, *B*. *bassiana* stands out among the entomopathogenic fungi, thus making it an attractive and widely used biopesticide against a lot of arthropod hosts.

Lastly, it is good to note that the complex mechanism of pathogenesis exhibited by *B*. *bassiana* cannot be fully understood by a singular omics approach, there is a need to examine the total expressions of different proteins, secondary metabolites, and their genes at every infection stage. Hence, researchers in different fields of omics need to collaborate to work with the same parameters to have a holistic view of the mechanism of action of different biopesticides.

## 4. Integrated pest management system

Integrated Pest Management (IPM) system refers to the mechanism of controlling pests using different techniques, such as habitat manipulation, biological and chemical control measures, use of pest-resistant varieties, and the modification of cultural practices. These techniques can be merged to ensure the long-term protection of plants (Deguine et al., 2021). For instance, IPM has been used in the control of *Tuta absoluta*, a deadly pest that affects tomatoes globally, and has developed resistance to insecticides (Desneux et al., 2021). Here, the synthetic pesticides and biological pesticides include the release and conservation of sex pheromones and arthropod natural enemies (Desneux et al., 2021). The use of IPM has been reported to be cost-effective and reduces the loss of crop yield (Hagstrum and Flinn, 2018). Currently, the adoption of IPM is limited owing to several factors, which include awareness, user preference, production industry, technology, policy, and culture (Deguine et al., 2021). It is, therefore, necessary to increase awareness of the inclusion of biological pesticides from microorganisms, plants, and nanobiopesticides in IPM. The awareness of many people about IPM will be an advantage to encourage producers to produce more of it, enhance its adoption and encourage researchers to carry out more research on it.

## 5. Future prospects and conclusion

A lot of crops are lost yearly to pest, but the emergence of synthetic pesticides have helped to reduce the loss. Nevertheless, the adverse effects of synthetic pesticides limit their use; thus, promoting the use of biological pesticides. Since biopesticides have proven as good alternative to chemical pesticides, it will be very important to explore them for maximum use in agriculture. The demand and availability of biopesticides are very poor, hence discouraging the producers and the users, respectively. Therefore, making grants or capital available for researchers, entrepreneurs, producers, and marketers will help to enhance the production and availability of biopesticides.

The shelf-life of biopesticides is short, as they require special temperatures and conditions for survival during transportation and storage. Hence, more research to unravel the mechanisms to make biopesticides more stable and improve their shelf-life will go a long way in increasing their efficiency.

The fact that biopesticides have no residual effects on the environment could serve as an advantage and a disadvantage. An advantage because it will not remain long enough to be dangerous to the plants, humans, and animals (which is one of the major demerits of synthetic pesticides), and it is a disadvantage because it will only protect crops as long as it has contacts with the pests, and pests that infest after their application would not be affected and might need another application, thus leading to a higher cost implication and labor for farmers. Consequently, more research should be carried out to incorporate bio-carriers and other sustainable methods, which can be used to enhance the persistence of biopesticides in the environment. Since biopesticides are highly specific in their mode of action, chemical reactions may occur if more than a biopesticide is applied to a crop that is affected by different pests. Hence, it is important to carry out more research on the compatibility of different biopesticides, which are likely to be used together on the same crop. Furthermore, most research carried out on biopesticides was focused on yield and not the nutritional quality of the crops, an insight into the nutritional quality of biopesticides will enhance their use.

The Maximum Residual Limit for pesticides in local markets (not only for food crops that would be exported) should be enforced and awareness should be created on the effectiveness of biopesticides so that farmers can explore them. In addition, a mobile meter, device, or strip could be developed, made affordable and easily available to enhance the easy and rapid detection of pesticide levels in food crops. This will help farmers to take caution and also help the populace to avoid feeding on crops with a Maximum Residual Limit greater than the WHO specified value. Awareness of the effects of the indiscriminate use and health effects of biopesticides in humans will also help to promote a good environment and health. Due to the numerous challenges still encountered with the use of biopesticides, the sole use of biopesticides might not be feasible. Therefore, their incorporation with the existing synthetic pesticides will be a better means of preventing crops from pests and ensuring sustainable agriculture.

## Author contributions

MA, BA, and SA conceived the idea and were involved in the writing of the manuscript. CF, UA, LG, RO, RJ, and QU contributed to the writing of the manuscript. MA, BA, and RO revised the manuscript. OB reviewed and edited the final draft of the manuscript. All authors contributed to the article and approved the submitted version.

## Funding

This study was funded through research grants from the National Research Foundation, South Africa (UID: 123634 and 132595).

## Conflict of interest

The authors declare that the research was conducted in the absence of any commercial or financial relationships that could be construed as a potential conflict of interest.

## Publisher’s note

All claims expressed in this article are solely those of the authors and do not necessarily represent those of their affiliated organizations, or those of the publisher, the editors and the reviewers. Any product that may be evaluated in this article, or claim that may be made by its manufacturer, is not guaranteed or endorsed by the publisher.
