# Corrigendum: Biopesticides as an alternative to synthetic pesticides: a case for nanopesticides, phytopesticides and microbial pesticides

**DOI:** 10.3389/fmicb.2023.1258968

**Published:** 2024-01-03

**Authors:** Modupe S. Ayilara, Bartholomew S. Adeleke, Saheed A. Akinola, Chris A. Fayose, Uswat T. Adeyemi, Lanre A. Gbadegesin, Richard K. Omole, Remilekun M. Johnson, Qudus O. Uthman, Olubukola O. Babalola

**Affiliations:** ^1^Food Security and Safety Focus Area, Faculty of Natural and Agricultural Sciences, North-West University, Mmabatho, South Africa; ^2^Department of Biological Sciences, Kings University, Ode-Omu, Nigeria; ^3^Department of Biological Sciences, Microbiology Unit, School of Science, Olusegun Agagu University of Science and Technology, Okitipupa, Nigeria; ^4^Department of Microbiology and Parasitology, School of Medicine and Pharmacy, College of Medicine and Health Sciences, University of Rwanda, Butare, Rwanda; ^5^Department of Agricultural Technology, Ekiti State Polytechnic, Isan-Ekiti, Nigeria; ^6^Department of Agricultural Economics and Farm Management, Faculty of Agriculture, University of Ilorin, Ilorin, Nigeria; ^7^Institute of Mountain Hazards and Environment, University of Chinese Academy of Sciences, Chengdu, China; ^8^Department of Microbiology, Obafemi Awolowo University, Ile-Ife, Nigeria; ^9^Microbiology Unit, Department of Applied Sciences, Osun State College of Technology, Esa-Oke, Nigeria; ^10^Soil, Water and Ecosystem Sciences, University of Florida, Gainesville, FL, United States

**Keywords:** nanoparticles, biopesticides, synthetic pesticides, soil health, pesticides

In the published article, there was an error with Figure 1. There was a close similarity between Figure 1 and a previously published Figure. The corrected Figure 1 and its caption appear below.

**Figure 1:**
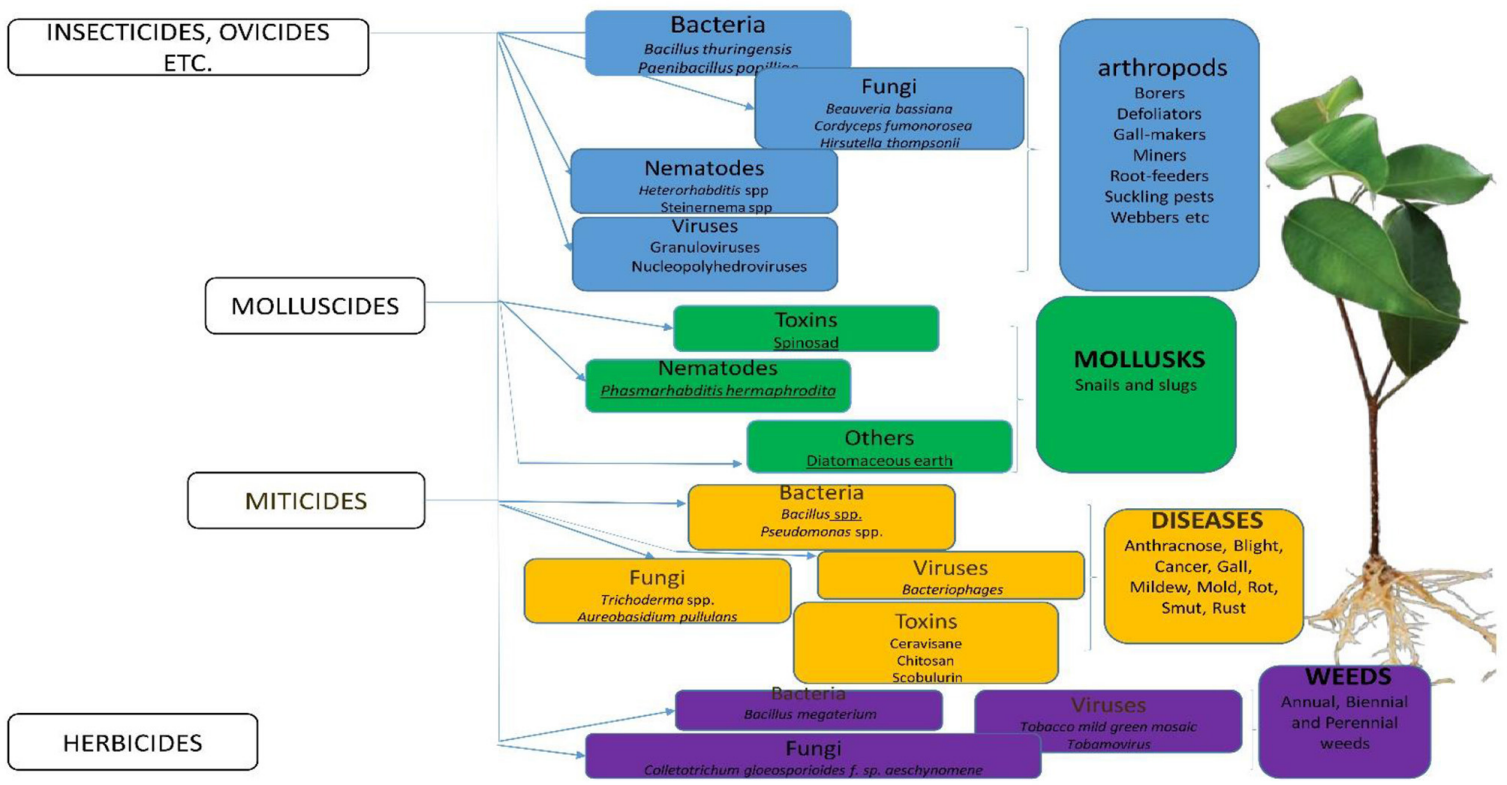
Classification of bio-pesticides and their target pests.

The authors apologize for this error and state that this does not change the scientific conclusions of the article in any way. The original article has been updated.

